# Contrasting plant transcriptome responses between a pierce-sucking and a chewing herbivore go beyond the infestation site

**DOI:** 10.1186/s12870-024-04806-1

**Published:** 2024-02-19

**Authors:** Álvaro Montesinos, Soledad Sacristán, Palmira del Prado-Polonio, Ana Arnaiz, Sandra Díaz-González, Isabel Diaz, M. Estrella Santamaria

**Affiliations:** 1 Centro de Biotecnología y Genómica de Plantas, Universidad Politécnica de Madrid (UPM) – Instituto Nacional de Investigación y Tecnología Agraria y Alimentaria (INIA/CSIC) Campus de Montegancedo, Pozuelo de Alarcón, 28223 Madrid, Spain; 2 Universidad de Zaragoza, Calle Pedro Cerbuna, 12, Zaragoza, 50009 Spain; 3 Departamento de Biotecnología-Biología Vegetal, Escuela Técnica Superior de Ingeniería Agronómica, Alimentaria y de Biosistemas, Universidad Politécnica de Madrid, Madrid, Spain; 4 Present Address: Departamento de Química, Facultad de Ciencias, Universidad de Burgos, Plaza de Misael Bañuelos s/n, Burgos, 09001 Spain

**Keywords:** *Arabidopsis thaliana*, Leaves, *Pieris brassicae*, Phytophagous infestation, Roots, *Tetranychus urticae*

## Abstract

**Background:**

Plants have acquired a repertoire of mechanisms to combat biotic stressors, which may vary depending on the feeding strategies of herbivores and the plant species. Hormonal regulation crucially modulates this malleable defense response. Jasmonic acid (JA) and salicylic acid (SA) stand out as pivotal regulators of defense, while other hormones like abscisic acid (ABA), ethylene (ET), gibberellic acid (GA) or auxin also play a role in modulating plant-pest interactions. The plant defense response has been described to elicit effects in distal tissues, whereby aboveground herbivory can influence belowground response, and vice versa. This impact on distal tissues may be contingent upon the feeding guild, even affecting both the recovery of infested tissues and those that have not suffered active infestation.

**Results:**

To study how phytophagous with distinct feeding strategies may differently trigger the plant defense response during and after infestation in both infested and distal tissues, *Arabidopsis thaliana* L. rosettes were infested separately with the chewing herbivore *Pieris brassicae* L. and the piercing-sucker *Tetranychus urticae* Koch. Moderate infestation conditions were selected for both pests, though no quantitative control of damage levels was carried out. Feeding mode did distinctly influence the transcriptomic response of the plant under these conditions. Though overall affected processes were similar under either infestation, their magnitude differed significantly. Plants infested with *P. brassicae* exhibited a short-term response, involving stress-related genes, JA and ABA regulation and suppressing growth-related genes. In contrast, *T. urticae* elicited a longer transcriptomic response in plants, albeit with a lower degree of differential expression, in particular influencing SA regulation. These distinct defense responses transcended beyond infestation and through the roots, where hormonal response, flavonoid regulation or cell wall reorganization were differentially affected.

**Conclusion:**

These outcomes confirm that the existent divergent transcriptomic responses elicited by herbivores employing distinct feeding strategies possess the capacity to extend beyond infestation and even affect tissues that have not been directly infested. This remarks the importance of considering the entire plant’s response to localized biotic stresses.

**Supplementary Information:**

The online version contains supplementary material available at 10.1186/s12870-024-04806-1.

## Background

Throughout their life cycle, plants encounter diverse environmental cues, including abiotic and biotic stresses. As sessile organisms, plants have developed diverse mechanisms to withstand pathogens infections and/or phytophagous infestations [1]. Depending on the pest feeding mode, plant response can vary, activating specific and complex mechanisms to combat the infestation [2]. Though severity of infestation is a major determinant of tissue damage, phloem-feeding or piercing-sucking herbivores typical infestation conditions cause reduced direct damage to plant tissues, resulting in fewer changes in the plant transcriptome in these instances. In contrast, chewing herbivores inflict a substantial physical damage in moderate infestations which leads to more pronounced alterations in the transcriptome profile, affecting multiple biological processes [2, 3].

Once an herbivore successfully bypasses the physical barriers of a plant, molecular plant responses are triggered to defend against the attack. Located on the plant’s surface, pattern recognition receptors (PRRs) recognize specific molecules known as herbivore-associated molecular patterns (HAMPs), or damage-associated molecular patterns (DAMPs). This produces a cascade of signaling events that results in differential hormonal regulation, and the biosynthesis of defense compounds in the plant [1, 4–6]. Hormonal regulation of plant defense is driven by two key regulators, jasmonic acid (JA) and salicylic acid (SA), with the former acting as a positive regulator of immunity against necrotrophic pathogens and chewing-biting herbivores, and the latter being predominantly involved in defense against biotrophic pathogens and piercing-sucking phytophagous while also being critical for induced and long-lasting resistance [2]. Besides, other hormones such as abscisic acid (ABA), ethylene (ET), gibberellins (GAs) and cytokinins (CK) are also involved in the intricate hormonal crosstalk that regulates plant immunity [1, 4–6]. Furthermore, auxin, primarily known for its role in plant growth regulation, has recently been suggested to have an additional role in plant defense [5, 7].

The plant defensive response acts at the site of infestation, but it generally has effects on the plant as a whole [8–10], mediated by long distance signals that move systemically through the plant. In addition, this response incites phytohormone mediated resistance trade-offs, which may have lasting consequences in the plant development even after infestation [11]. Extensive research has been conducted to investigate the reciprocal effects of aboveground herbivory on belowground infestation and vice versa, demonstrating the detrimental impact of infestation in a tissue to distal tissues plagues [12–15]. Thus, pest attack to the leaves may have consequences on distal tissues, such as the root, which plays a crucial part in water and nutrient uptake and have a critical role in the interaction between the plant and its environment, including other organisms [16, 17]. Infestation might disrupt these essential processes, which are vital for optimal plant development [18]. However, the characterization of molecular responses in roots influenced by distal infestation has been relatively limited [19, 20]. While some studies have examined the influence of aboveground herbivory on the production of root defense-related compounds [21, 22], only a few have reported on the specific effects on the transcriptome [23, 24], and none with cell content feeders. Besides, the lasting effects in root development of pest leaf attack in the post-infestation recovery are yet to be thoroughly characterized.

In this study, we compare the plant response of *Arabidopsis thaliana* L. to two phytophagous pests, *Pieris brassicae* L. and *Tetranychus urticae* Koch, which exhibit distinct feeding modes on leaves. *P. brassicae* is a chewing leaf-feeder caterpillar, while *T. urticae*, commonly known as two spotted spider mite, is a piercing-sucking pest. The molecular response of leaves and roots was analyzed at two different times: after 24 h of infestation (collection time 24 h) and 48 h after the removal of the phytophagous (collection time 72 h). Our findings reveal distinct responses to moderate infestation by *P. brassicae* and *T. urticae* that extend to the roots and persist beyond the period of infestation.

## Materials and methods

### Plant material and growth conditions

*Arabidopsis thaliana* L. Col-0 accession was used (Nottingham Arabidopsis Seed Collection). Seeds were planted and stratification was carried out 5 d in the dark at 4 °C in peat moss and vermiculite (3:2 V/V) in 70 mL pots. Plants were then grown in growth chambers (RADIBER Modelo AGP-1400) under control conditions (23 °C ± 1 °C, > 70% relative humidity, and a 16 h/8 h day/night photoperiod).

### Phytophagous maintenance

A colony of *T. urticae*, London strain (Acari: *Tetranychidae*) provided by Dr. Miodrag Grbic (UWO, Canada), was reared on beans (*Phaseolus vulgaris*) and maintained in a growth chamber (Sanyo MLR-350‐H) at 25 °C ± 1 °C, > 70% relative humidity and a 16 h/8 h day/night photoperiod. A *P. brassicae* colony supplied by Prof. Dr. Marcel Dicke and Dr. Pieter Rouweler (Laboratory of Entomology, Wageningen University, Netherlands), was reared on Brussels sprouts (*Brassica oleracea* L. var. *gemmifera*) and maintained on a growth chamber (Sanyo MLR-350-H, Sanyo, Japan) at 21ºC ± 1ºC, 50% relative humidity and a 16 h/8 h day/night photoperiod.

### Plant infestation assays

**Fig. 1:**
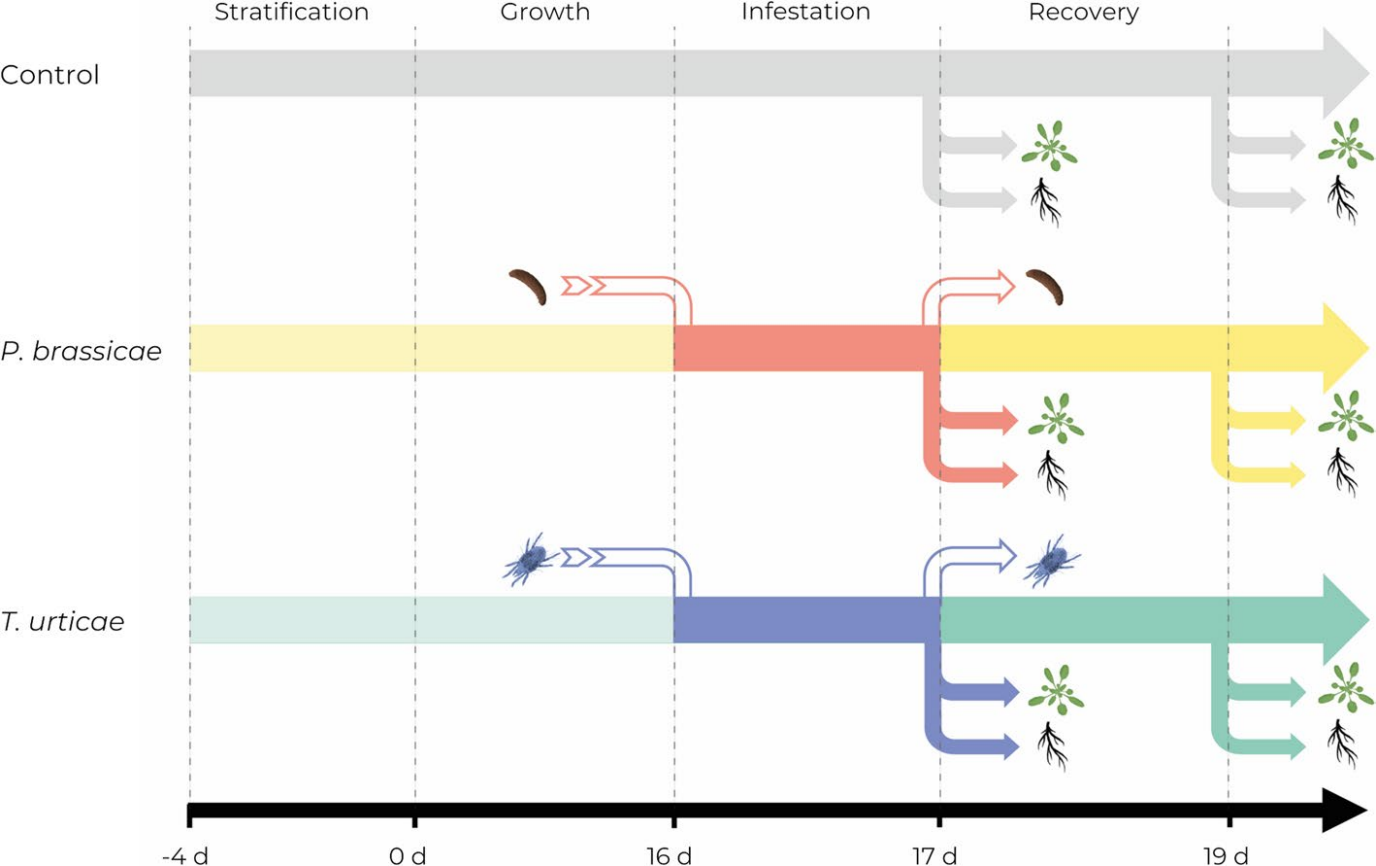
Scheme representing the experiments timeline. Plants were stratificated for 5 d and inoculated at 16 d after-germination. Phytophagous removal was carried out at 24 h post-infestation. Samples were collected at 24 and 72 h post-infestation

Sixteen-day-old plants were infested with 20 synchronized *T. urticae* adult female or 5 synchronized freshly neonate *P. brassicae* caterpillar per plant. Number of phytophagous was selected from a previous characterization to achieve a moderate infestation in both treatments [25]. They were carefully transferred with a brush to the leaf surface. To avoid herbivore escape and cross-infestation, individual plants were confined to a transparent cylinder with aeration. Plants were subject to two different treatment regimens: For 24 h treatment, plant material was harvested after 24 h of *T. urticae* or *P. brassicae* infestation. For 72 h treatment, phytophagous were removed after 24 h and plants were collected 48 h later (Fig. 1). Control plants did not contain any phytophagous and were sampled at the same time. Rosettes were harvested and collected in liquid nitrogen and stored at -80 ºC. Roots were carefully washed with tap water and excised from below the crown point, being posteriorly collected in liquid nitrogen and stored at -80 ºC. Three replicates of 4 plants each were performed per treatment and control. Plants for each treatment were placed randomized within the chamber.

### RNA-Seq library preparation, sequencing, alignment and DEG analysis

Total RNA was isolated and purified by using RNeasy Qiagen Mini Plant Kit (74904 Qiagen), including the on-column DNA I (79254, Qiagen) digestion recommended by the manufacturer. RNA amount and quality were tested in a Nanodrop ND‐1000.

RNA samples were sent for stranded mRNA-Seq analysis to Novogene (Novogene UK CL). Libraries preparations from mRNA were sequenced on an Illumina platform, generating over 30 M paired-end reads per sample. HISAT2 software [26] was used to align the paired-end clean reads to the *A. thaliana* reference genome (TAIR10; https://www.arabidopsis.org/). HTSeq [27] was utilized to quantify the read numbers mapped to each gene, and the reads per kilobase million (RPKM) for each gene was calculated. The differential expression analysis was performed using the DESeq2 R package [28]. The *p*-values resulting from this analysis were adjusted using the Benjamini and Hochberg’s correction. Genes with an adjusted *p*-value of less than 0.05 and a log_2_FC above 1 or below − 1 were considered to be differentially expressed.

### RNA-Seq data structural and functional analysis

Principal component analysis (PCA) was performed on gene expression values across all conditions using the R stats package with default parameters and visualized using the factoextra R package. Gene ontology (GO) enrichment analysis was conducted using the topGO R package [29]. A Fisher’s exact test was applied with a cut-off of *p*-value < 0.05. Significant GO terms were visualized using the GO-Figure! Python package [30]. KEGG Kyoto Encyclopedia of Genes and Genomes) [31–33] enrichment analysis was carried out using the edgeR R package [34–36], with a *p*-value cut-off of < 0.05. Lists of receptor and transcription factor (TF) genes were obtained from public repositories. The receptor gene list was built from two different platforms: the Plant Resistance Genes Database 3.0 and the resistance gene analogs (RGAs) lists of genes [37, 38]. TFs were downloaded from the Plant Transcription Factor Database (PlantTFDB v5.0) integrated in the PlantRegMap platform [39–41]. Heatmaps were generated using the gplots R package.

## Results

### RNA-Seq data structural analysis

**Fig. 2:**
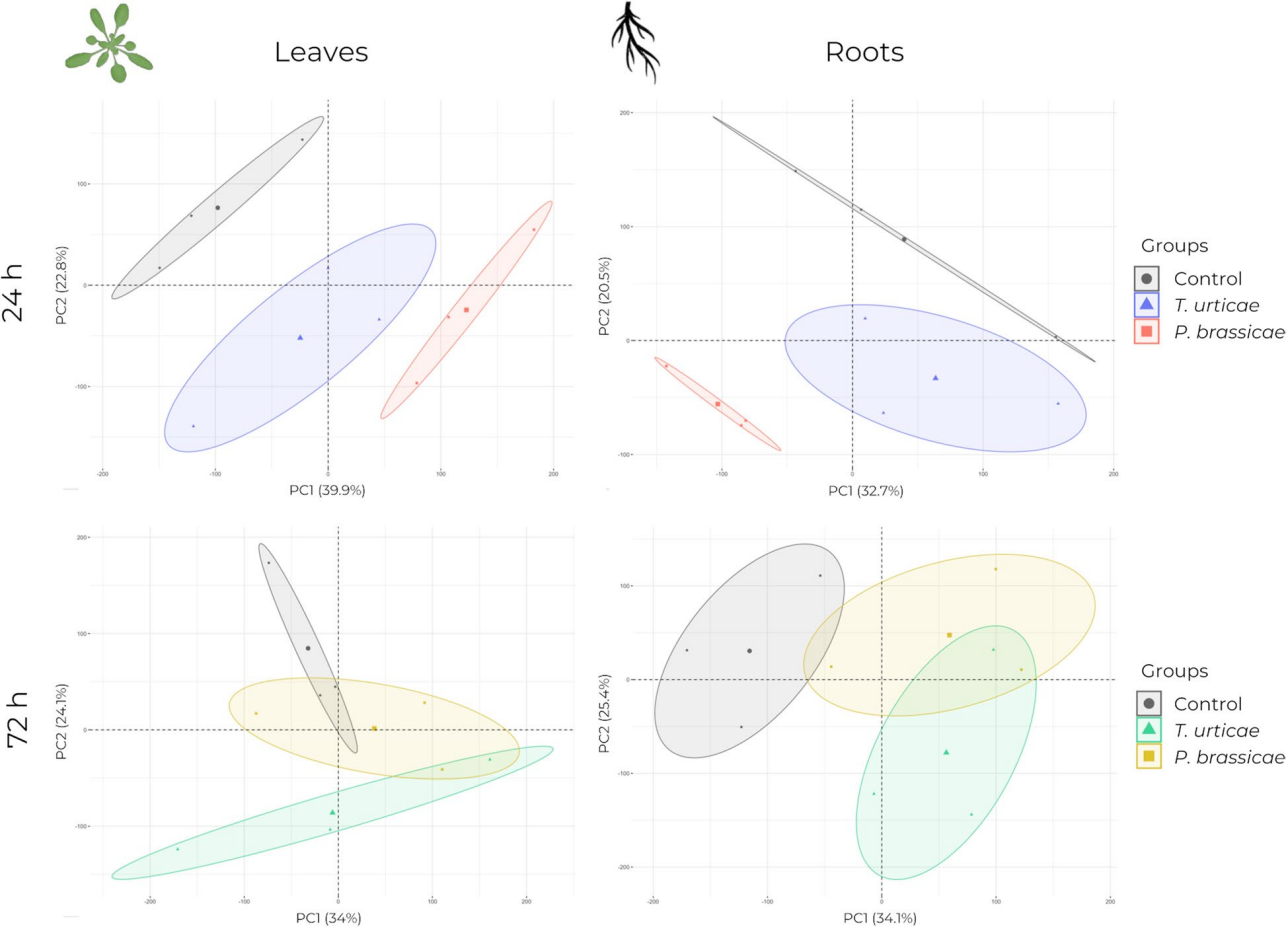
Principal component analysis (PCA) of the global expression profile data. Represented for the different tissues and their collection time categorized by phytophagous

A PCA was performed on the RNA-Seq data (Additional file 1) obtained from leaves and roots for both treatments, after 24 h of infestation and at 72 h, 48 h after phytophagous removal. The first two principal components explained a significant proportion of the variability in both tissues at 24 h (Leaves: PC1 = 39.9%, PC2 = 22.8%; Roots: PC1 = 32.7%, PC2 = 20.5%). No other components were found to account for more than 10% of the variability. Sample distribution was observed to be similar in both tissues at 24 h, with control plants, plants infested with *P. brassicae* and plants infested with *T. urticae* being clearly separated from each other in the PCA plot (Fig. 2). The plants infested with *P. brassicae* exhibited a greater separation from the control plants. At 72 h, the sample distribution was similar between leaves and roots, with the first components explaining almost 60% of the variability in both tissues (Leaves: PC1 = 34%, PC2 = 24.1%; Roots: PC1 = 34.1%, PC2 = 25.4). The samples from infested plants were found to be closer to those from control plants, indicating that there was more differential expression at 24 h (Fig. 2). In contrast to 24 h, the samples from plants infested with *T. urticae* were more separated from the control plants at 72 h than samples from plants infested with *P. brassicae* (Fig. 2).

**Fig. 3:**
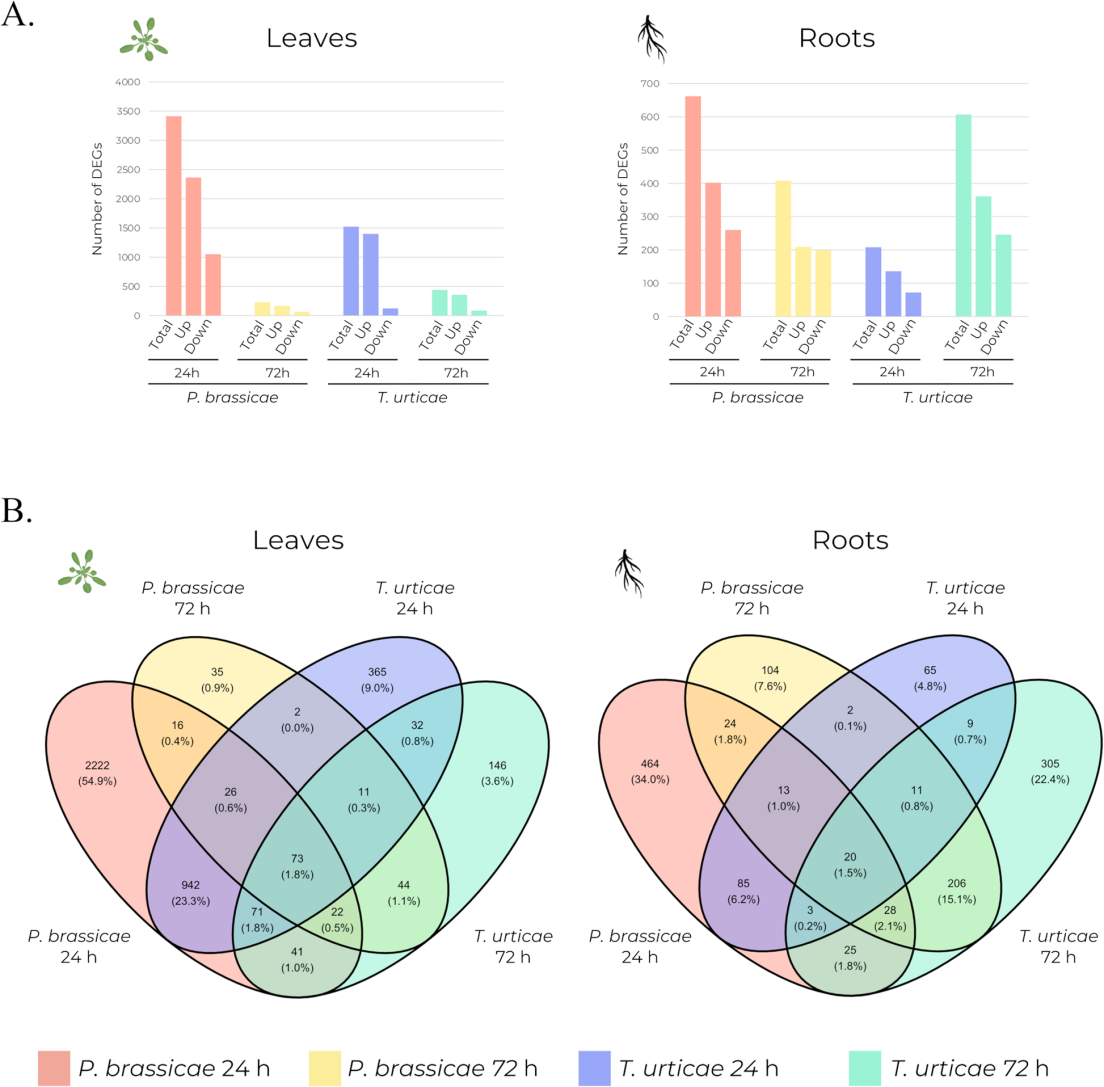
Number of DEGs through all treatments. **a** Number of total, upregulated and downregulated DEGs in leaf and root tissues at 24 and 72 h post-infestation by *P. brassicae* and *T. urticae*. **b** Venn diagrams of DEGs in leaf and root tissues at 24 and 72 h post-infestation by *P. brassicae* and *T. urticae*

A total of 3,413 differentially expressed genes (DEGs) were identified at 24 h in leaves infested with *P. brassicae*, with 2,363 upregulated and 1,050 downregulated. At 72 h, the number of DEGs drastically decreased to 229 (165 upregulated and 64 downregulated) DEGs (Fig. 3a). A similar trend was observed in leaves infested with *T. urticae*, with 1,522 (1,399 upregulated and 123 downregulated) DEGs at 24 h and 440 (355 upregulated and 85 downregulated) DEGs at 72 h (Fig. 3a). However, the drop in DEGs after plant recovery in leaves was less pronounced in *T. urticae* than in *P. brassicae*. In roots, there was a different tendency between plants infested with *P. brassicae* and *T. urticae*. Although the number of DEGs was lower compared to leaves at 24 h, root samples from *P. brassicae* infested plants presented a similar profile. At 24 h roots showed 662 (402 upregulated and 260 downregulated) DEGs, while at 72 h, the number decreased to 408 (209 upregulated and 199 downregulated) DEGs (Fig. 3a). Plants infested with *T. urticae* showed a contrasting profile, with 208 (136 upregulated and 72 downregulated) DEGs at 24 h and 607 (361 upregulated and 246 downregulated) DEGs at 72 h (Fig. 3a). In general, the response to both phytophagous was mostly upregulated, but this was especially patent in leaves exposed to *T. urticae*, where 91% and 81% of the DEGs were upregulated at 24 and 72 h respectively. In the roots at 72 h, however, the proportion of up and downregulated genes was more balanced, with 51% and 59% of upregulated genes in *P. brassicae* and *T. urticae* respectively (Fig. 3a).

The plant responses exhibited a certain degree of overlap between the two phytophagous. In leaves, approximately 30% of the DEGs were found to be common between plants infested with *P. brassicae* and *T. urticae* at both 24 and 72 h treatment (Fig. 3b). However, in roots, the limited number of DEGs at 24 h resulted in a smaller overlap between the phytophagous, whereas at 72 h, the overlap was again above 30% (Fig. 3b). Nevertheless, the DEGs exhibited notable differences when comparing treatments or tissues (Additional file 2). The most exclusive response was that of leaves and roots after 24 h of exposure to *P. brassicae*, where 65% and 70% of the DEGs, respectively, were not shared by other samples (Fig. 3b). This situation changed dramatically at 72 h, where there were just 15% of DEGs exclusive of leaves and 25% of DEGs exclusive of roots. In the case of leaves at 72 h, most of the DEGs in *P. brassicae* overlapped with DEGs at 24 h, or 72 h in *T. urticae* almost indistinctly (61% or 66%, respectively). In the case of roots at 72 h, the response was clearly shared with that of *T. urticae* (65% of DEGs). In the case of *T. urticae*, the exclusive response was around 24–33% of DEGs in all cases, except at 72 h in the roots, where it increased to 50% of DEGS (Fig. 3b).

### RNA-Seq data functional analysis

**Fig. 4:**
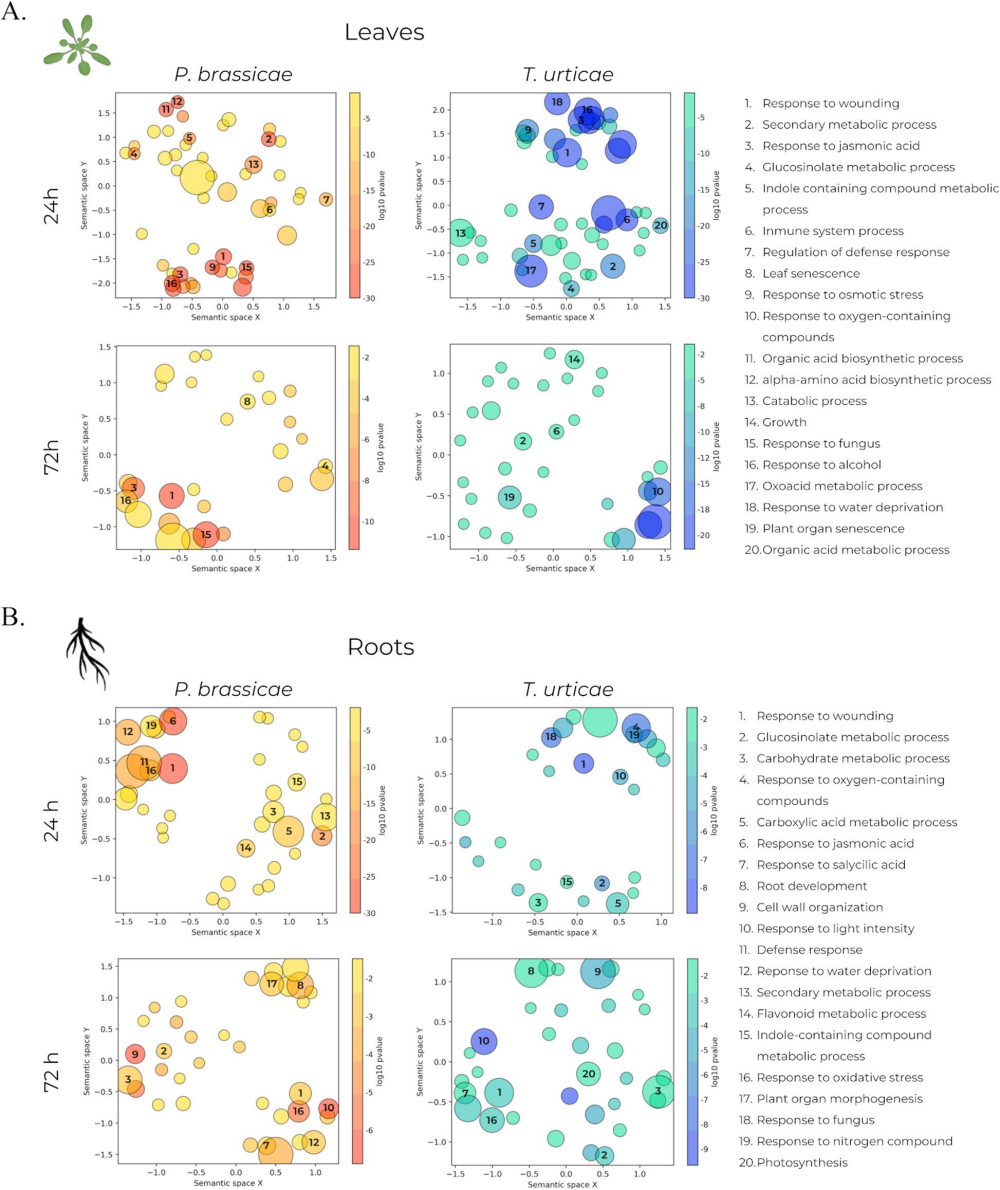
Representation of relevant GO terms constructed using GO-Figure! [30]. GO terms are filtered and grouped by semantic similarity. Bubble size represents number of genes associated to each term. All GO terms represented in the figure can be found in Additional file 3. **a** GO terms enriched in leaves at 24 and 72 h post-infestation by *P. brassicae* and *T. urticae*. **b** GO terms enriched in roots at 24 and 72 h post-infestation by *P. brassicae* and *T. urticae*

An enrichment analysis using Gene Ontology (GO) was carried out to characterize the biological processes involved in the plant response to infestation by *P. brassicae* and *T. urticae*. The resulting data (Additional file 3) was visualized using the GO-Figure! Python package [30] to facilitate the identification of shared processes between different conditions. At 24 h, leaves infested with either phytophagous displayed an identifiable group of GO terms associated with stress and defense responses, including response to wounding, JA, alcohol, osmotic stress, and water deprivation (Fig. 4a). Isolated from these are other terms involved in defense processes, like immune system process and regulation of defense response. Additionally, there was an identifiable group of GO terms related to secondary metabolic process and glucosinolate and amino acid biosynthesis, which are relevant plant defense compounds (Fig. 4a). At 72 h, only plants that had been infested with *P. brassicae* showed a group of enriched stress response terms. Terms related to secondary metabolism and senescence, such as plant organ or leaf senescence, were identified in both *P. brassicae* and *T. urticae* infested plants (Fig. 4a).

In the case of both phytophagous infestations, roots presented a similar display than leaves at 24 h. Two distinct groups of enriched terms could be identified. The first one consisting of multiple terms related to stress response, such as response to wounding, JA, oxidative stress, and water deprivation. The second group was integrated by terms related to secondary metabolism, such as carboxylic acid, indole containing compound, glucosinolate, and flavonoid metabolic processes (Fig. 4b). At 72 h, there were differences in the GO terms categories observed between leaves and roots, with terms related to growth processes being enriched in roots, including root development, cell wall organization, and plant organ morphogenesis. Furthermore, root samples from plants infested with *T. urticae* displayed an enrichment of terms involving light perception or photosynthesis (Fig. 4b).

**Fig. 5:**
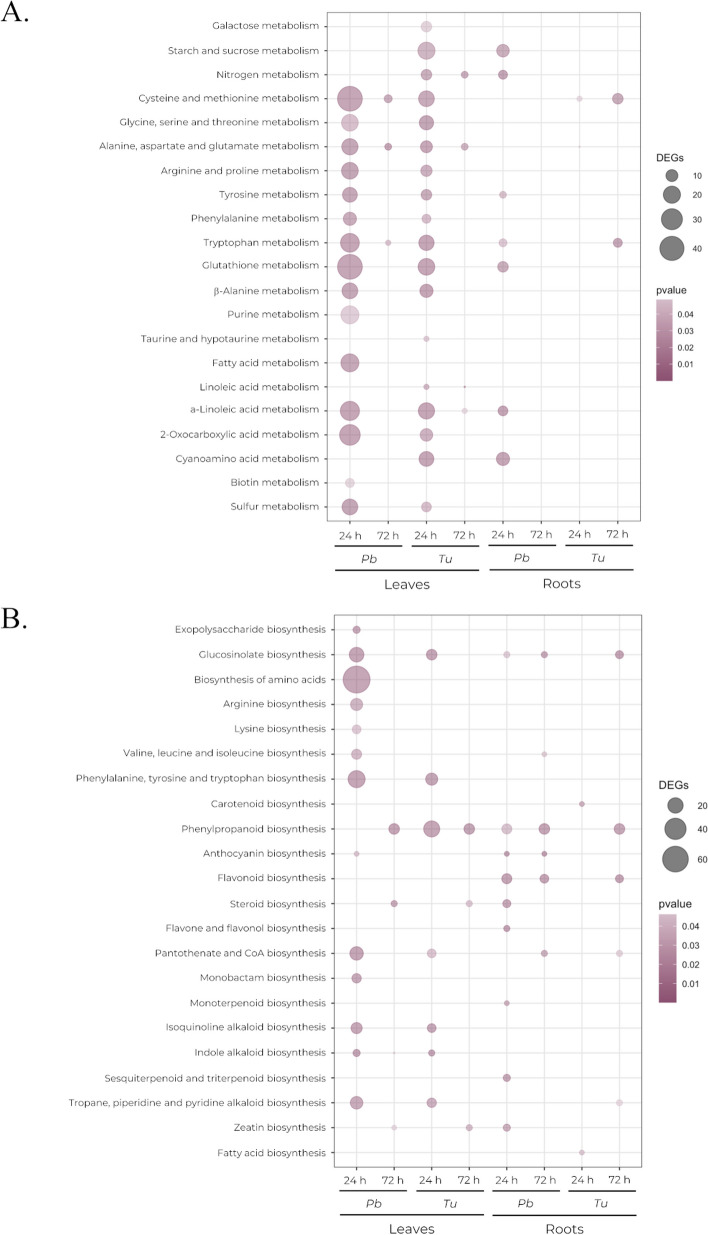
KEGG pathway [30–32] enrichment of DEGs in leaf and root tissues at 24 and 72 h post-infestation by *P. brassicae* (*Pb*) and *T. urticae (Tu*). **a** KEGG pathways associated with metabolism enriched for at least one condition. **b** KEGG pathways associated with biosynthesis enriched for at least one condition

Both *P. brassicae* and *T. urticae* infested plants presented an enrichment of KEGG pathways [31–33] related to amino acid metabolism, several of them involved in hormone biosynthesis, in leaves at 24 h. Pathways associated with defense compounds like glucosinolate biosynthesis or glutathione metabolism were also enriched at 24 h, while amino acid biosynthesis was only enriched in leaves infested with *P. brassicae* (Fig. 5a, b). Root samples from infested plants presented an enrichment of the biosynthesis of stress regulators such as flavonoids or anthocyanins, including the phenylpropanoid pathway, which may also affect the integrity of the cell wall (Fig. 5b).

### Phytophagous effect in plant hormonal response

Hormonal regulation is an essential component of the plant response to biotic stresses. The gene expression profile of several hormones linked to defense and stress response processes was examined. DEGs associated with hormones were identified through GO categorization and heatmaps were generated to visualize the DEGs found in at least one treatment.

**Fig. 6:**
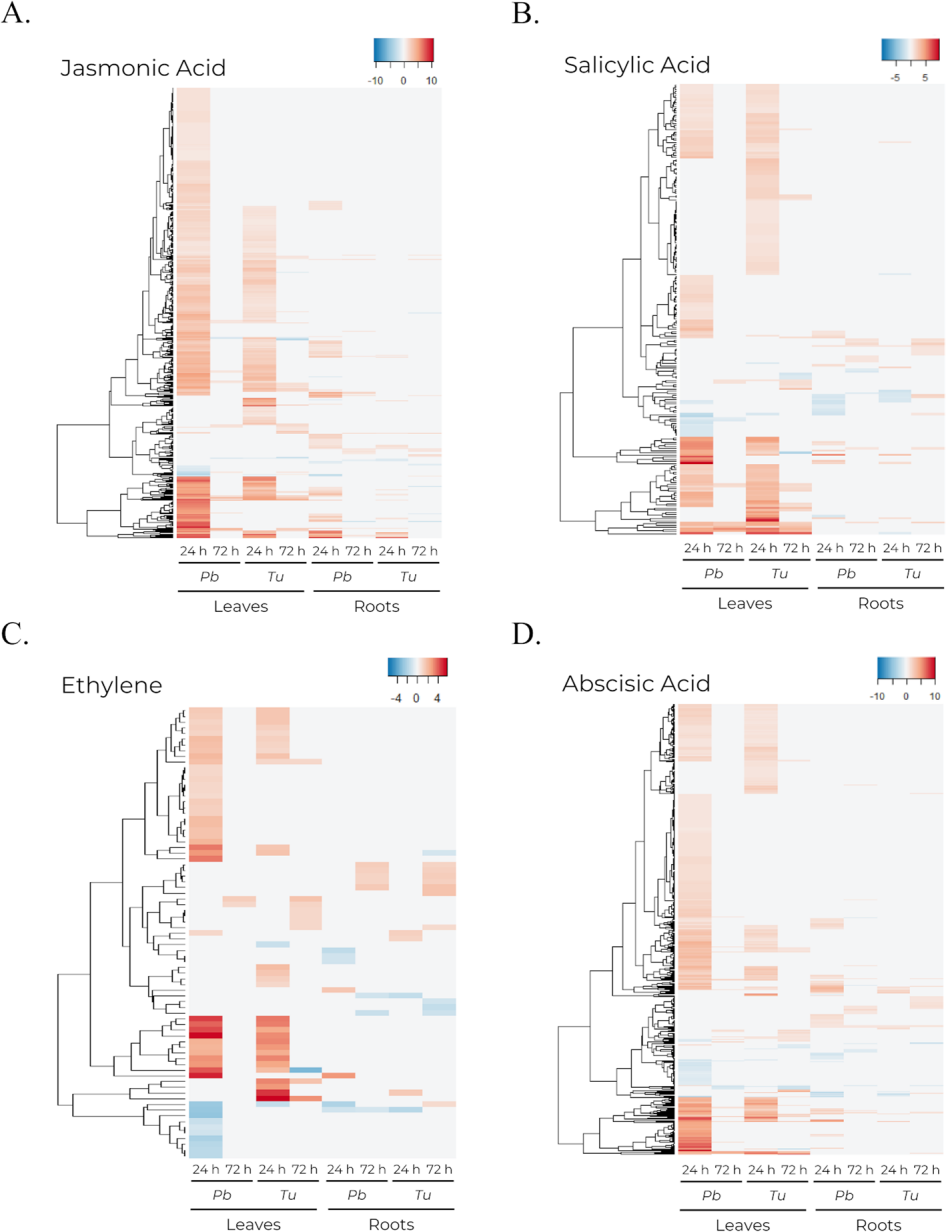
Heatmaps showing the transcriptomic profile of DEGs related to hormones. Comprises DEGs from leaf and root tissues at 24 and 72 h post-infestation by *P. brassicae* (*Pb*) and *T. urticae (Tu*) detected at least in one condition. **a** DEGs with at least one annotated GO term related to jasmonic acid. **b** DEGs with at least one annotated GO term related to salicylic acid. **c** DEGs with at least one annotated GO term related to ethylene. **d** DEGs with at least one annotated GO term related to abscisic acid

Expression profile of genes associated with JA was similar between leaves infested with either *P. brassicae* or *T. urticae* (Fig. 6a). Genes involved in defense or JA response, including *LOX2* or the *JAZ* gene family, exhibited a similar pattern of expression under both phytophagous. Meanwhile, genes involved in JA biosynthesis and modification were more expressed at 24 h in leaves infested with *P. brassicae* than with *T. urticae*. This group includes *AOC* genes, *JMT*, *JAR*, or *LOX3* and *LOX4*. Similarly, JA related genes involved in cell wall formation or stress response were upregulated at 24 h under both phytophagous, with higher expression levels observed in *P. brassicae* infested plants (Additional file 4). In roots, *P. brassicae* infested plants presented higher levels of upregulation at 24 h, though few DEGs related to JA were observed overall (Fig. 6a). Differently, at 24 h, SA associated DEGs were detected in leaves under both phytophagous, but plants infested with *T. urticae* displayed a higher degree of differential expression (Fig. 6b). This difference was more evident in SA related genes involved in defense response, whereas genes involved in cell wall formation or stress response had a similar expression in leaves from *P. brassicae* or *T. urticae* infested plants (Additional file 4). Differential expression in roots was reduced for all conditions (Fig. 6b).

In contrast to the SA-related expression profile, genes associated with ET were almost equally affected by either phytophagous in leaves, although more DEGs were detected when infested with *P. brassicae*, including genes involved in ET biosynthesis like *ACS2*, *ACS4* and *MKK9* (Additional file 4). Roots also presented a reduced number of DEGs related to ET (Fig. 6c). Finally, genes associated with ABA were upregulated in leaves at 24 h upon both infestations, with more DEGs reported when plants were infested with *P. brassicae* (Fig. 6d). This was visible for ABA associated genes involved in cell wall formation or reorganization. A similar trend was observed for genes involved in response to stress (Additional file 4). In roots, differential expression was limited, with samples collected at 24 h from plants infested with *P. brassicae* presenting a higher number of DEGs (Fig. 6d).

**Fig. 7:**
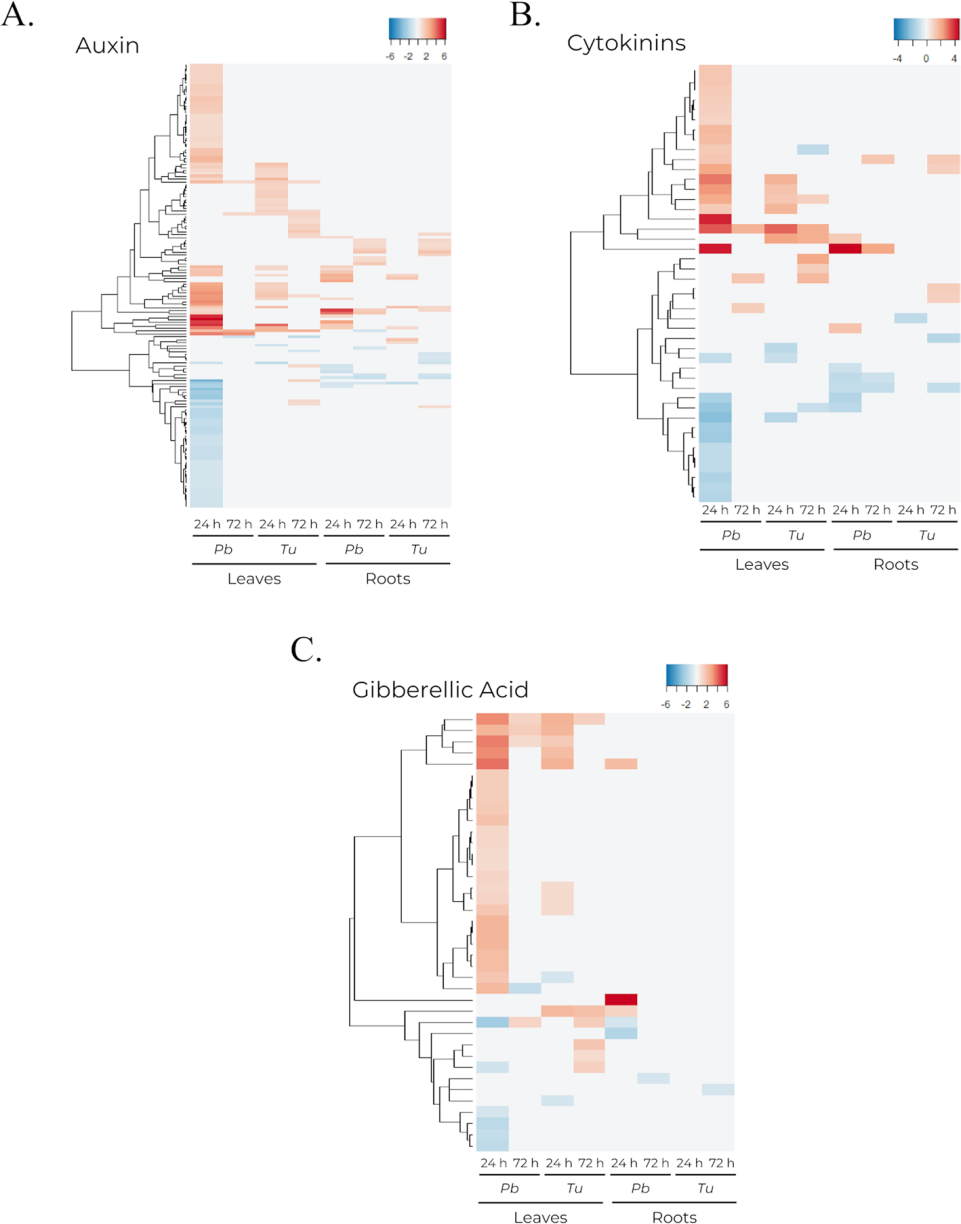
Heatmaps showing the transcriptomic profile of DEGs related to hormones. Comprises DEGs from leaf and root tissues at 24 and 72 h post-infestation by *P. brassicae* (*Pb*) and *T. urticae (Tu*) detected at least in one condition. **a** DEGs with at least one annotated GO term related to auxin. **b** DEGs with at least one annotated GO term related to cytokinin. **c** DEGs with at least one annotated GO term related to gibberellic acid

Leaves from *P. brassicae* infested plants displayed more DEGs associated with auxins (Fig. 7a). Genes involved in auxin availability and biosynthesis were overexpressed at 24 h under both phytophagous, but expression levels were higher in leaves infested with *P. brassicae*. This contrast was more evident regarding genes involved in auxin response (Additional file 4). Few DEGs were found in leaves infested with *T. urticae*, and only leaves from *P. brassicae* infested plants presented downregulated genes (Fig. 7a), including multiple members of the SAUR family of auxin responsive proteins. Moreover, genes involved in auxin transport only displayed differential expression under this condition (Additional file 4). Although the number of DEGs was significantly reduced in roots, the difference between both tissues was smaller for auxin-related genes, and the number of DEGs was consistent for both treatments in roots (Fig. 7a). Other growth-related hormones, such as CKs and GAs, exhibited a reduced number of DEGs that were mostly identified at 24 h in leaves infested with *P. brassicae* (Fig. 7b, c).

### Phytophagous effect in other processes involved in response to stress

**Fig. 8:**
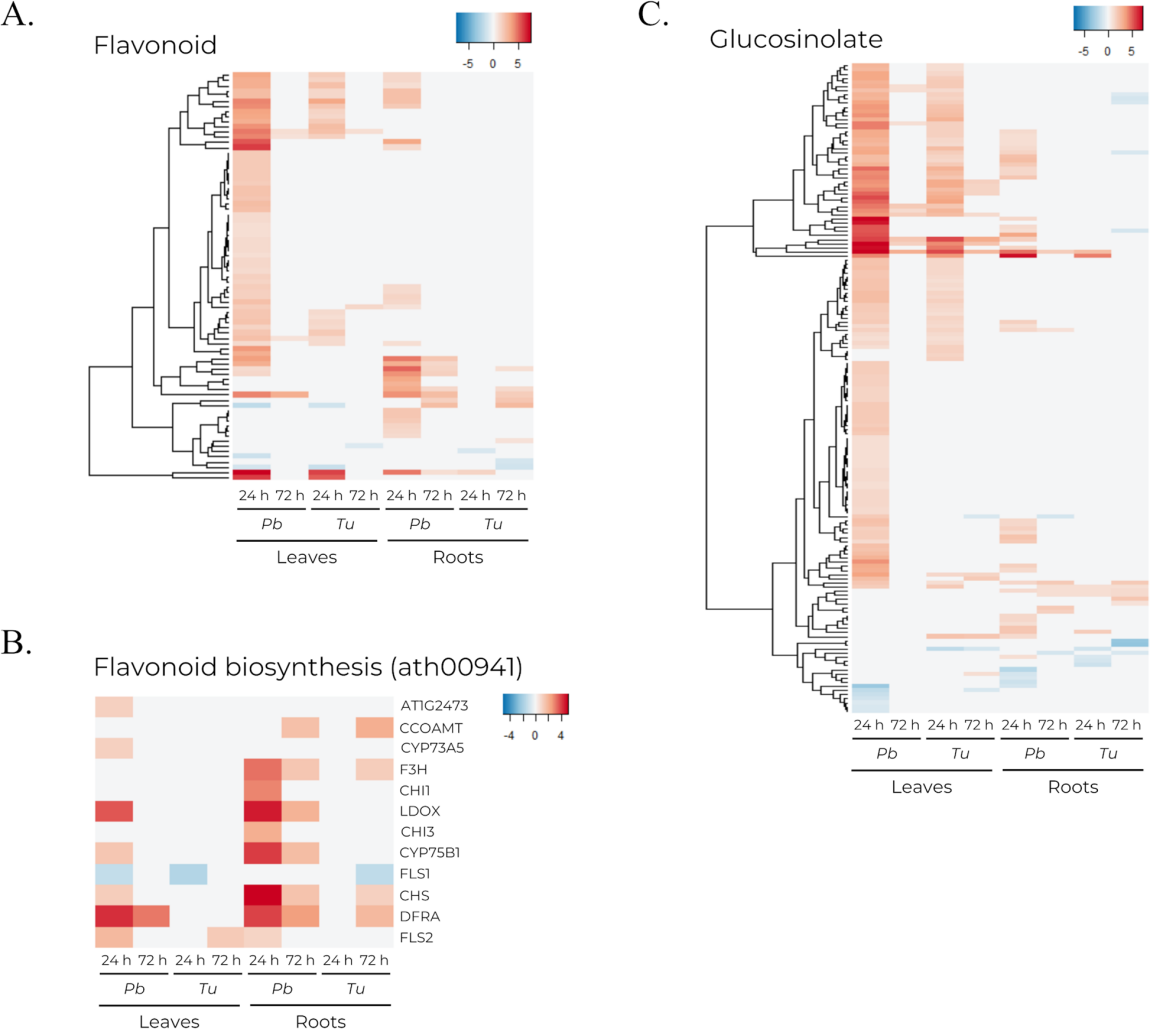
Heatmaps showing the transcriptomic profile of DEGs related to flavonoids and glucosinolates. Comprises DEGs from leaf and root tissues at 24 and 72 h post-infestation by *P. brassicae* (*Pb*) and *T. urticae (Tu*) detected at least in one condition. **a** DEGs with at least one annotated GO term related to flavonoids. **b** DEGs annotated in the flavonoid biosynthesis (ath00941) KEGG pathway. **c** DEGs with at least one annotated GO term related to glucosilonate

Flavonoids are relevant compounds involved in multiple biological processes, including response to stress. In this study, DEGs associated with flavonoid biosynthesis and regulation were upregulated at 24 h in leaves infested with *P. brassicae*, but limited differential expression was detected in *T. urticae* infested leaves (Fig. 8a). Genes involved in flavonoid biosynthesis were also overexpressed at both 24 and 72 h in roots from plants infested with *P. brassicae*, although expression levels were higher under active infestation at 24 h (Fig. 8b). Certain genes such as *LDOX*, *CYP75B1* or *CHS* presented a higher expression in roots than in leaves. In contrast, *T. urticae* infested plants only showed an overexpression of flavonoid biosynthesis genes in roots at 72 h (Fig. 8b).

Taking in consideration that glucosilonates and its conjugates play crucial roles in plant defense mechanisms, the expression pattern of genes linked to glucosinolate pathways was analyzed. It was observed that both biosynthesis and modification associated genes were upregulated at 24 h in leaves infested with either phytophagous, but expression levels were higher under *P. brassicae* infestation. In contrast, few DEGs were detected in leaves at 72 h or in roots. Exceptionally, roots from *P. brassicae* infested plants showed a certain degree of overexpression at 24 h (Fig. 8c).

**Fig. 9:**
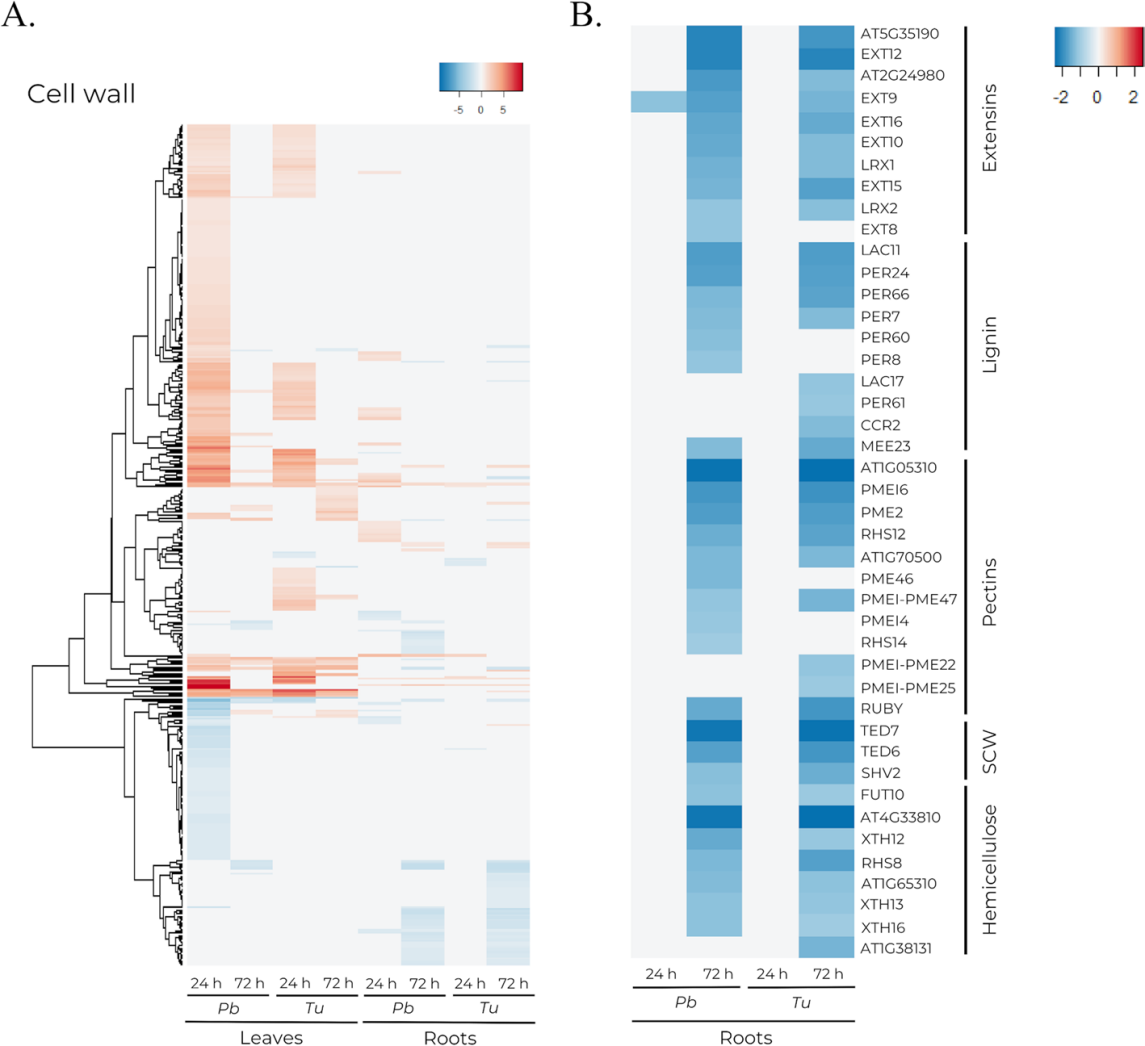
Heatmaps showing the transcriptomic profile of DEGs related to cell wall processes. Comprises DEGs from leaf and root tissues at 24 and 72 h post-infestation by *P. brassicae* (*Pb*) and *T. urticae (Tu*) detected at least in one condition. **a** DEGs with at least one annotated GO term related to cell wall processes. **b** Subset of DEGs with at least one annotated GO term related to cell wall processes downregulated in root tissue at 48 h after infestation

DEGs associated with cell wall formation or reorganization were identified at 24 h in leaves infested with either *P. brassicae* or *T. urticae* (Fig. 9a). Thus, genes participating in pectin regulation, including multiple pectinesterases (PME), were only upregulated at 24 h in leaves, whereas DEGs associated with lignin regulation extended their overexpression until 72 h. This includes various peroxidases. In contrast, genes related to hemicellulose regulation were differentially expressed mainly in leaves infested with *P. brassicae*, presenting at 24 h a mixed gene profile. Additionally, at 24 h, multiple expansins were downregulated in leaves infested by *P. brassicae*, while not being affected in *T. urticae* infested leaves (Additional file 4).

Roots from plants infested with either phytophagous presented multiple genes related to cell wall formation or reorganization downregulated at 72 h, including members of the extensin family, genes associated with lignin regulation such as peroxidases and laccases, PMEs, genes involved in hemicellulose regulation, and other genes related to the regulation of the secondary cell wall (Fig. 9b).

## Discussion

Herbivore infestation elicits multiple physiological and molecular alterations in the attacked tissues. Nevertheless, how infestation affects the regulation of molecular processes in distant plant parts, such as roots, remains largely uncharted. To answer this question, the transcriptomic profiles of leaves and roots from *A. thaliana* plants infested with either leaf phytophagous *P. brassicae* or *T. urticae* were analyzed. Differences observed in the transcriptomes due to their distinct feeding mode were not limited to the infestation site, but also extended to the root, indicating that the effects of infestation transcended the localized feeding zone in a feeding guild depending manner.

### Feeding mechanism differentially affects overall plant response to infestation

In this study, leaves and roots obtained from plants infested with *P. brassicae* exhibited a higher degree of divergency in their transcriptomic response than control plants (Fig. 2) and a higher number of DEGs compared to those infested with *T. urticae* after 24 h (Fig. 3a). Previous studies have demonstrated that the transcriptomic response to pests can vary depending on their feeding guild [2, 42]. For example, while leaf infestation by herbivores like the chewing caterpillar *Plutella xylostella*, can induce changes in the root transcriptome, others, such as the sucking aphid *Brevicoryne brassicae*, do not have a significant impact [23]. The higher physical damage caused to plant tissues by a moderate infestation of the chewing-biting herbivore *P. brassicae*, directly destroying foliar or flower tissues [43, 44], may account for the greater number of DEGs observed during active infestation by this pest in this instance in comparison to the piercing-sucking *T. urticae*, whose stylet penetrates through stomata or between pavement cells [45]. However, a reverse trend was observed when the stress had been removed, with plants previously infested by *T. urticae* exhibiting prolonged differential gene expression, indicating a difference in the timing of stress perception (Fig. 3a, b). In previous experiments, *T. urticae* induced a more robust activation of SA and immune response compared to the chewing-biting herbivore *Pieris rapae* [25], indicating a potentially significant role of induced resistance in the plant defense against *T. urticae*. In contrast, the response to *P. brassicae* may require rapid activation of stress response genes [46], which is asserted by the enrichment of GO categories like “response to oxidative stress” or “response to osmotic stress” (Additional file 3), promptly restoring a basal transcriptomic profile after infestation.

Although there were discernible differences in the transcriptomic responses to infestations by *P. brassicae* and *T. urticae*, a considerable number of DEGs were shared between the two infestations (Fig. 3b), indicating a similarity in the underlying biological processes that are involved during active infestation and after the removal of the phytophagous. This was also apparent regarding the enrichment of GO terms and KEGG pathways (Figs. 4 and 5). Plants exhibited enrichment of terms associated with response to stress and defense response in both tissues during active infestation (at collection time 24 h) and after phytophagous removal (at collection time 72 h). Specifically, infested leaves displayed an enrichment of terms related to secondary metabolism and pathways associated with amino acid metabolism, likely indicating a specific response active during the infestation, even affecting the biosynthesis of amino acids in leaves infested with *P. brassicae* (Fig. 5b) and arrested once the pests are removed. These are involved in the biosynthesis and modification of compounds relevant for plant defense [47–49].

However, when comparing different tissues or treatments, fewer DEGs were found to be shared (Additional file 2). This discrepancy suggests that leaves and roots present different gene expression in response to leaf infestation with the same phytophagous. Besides, the reduction in the number of DEGs between 24 and 72 h was more pronounced in leaves compared to roots (Fig. 3a). This may suggest that the response in leaves, where both herbivores are feeding, is immediate, whereas in roots, the response is somewhat delayed and active for a longer time. This trend is particularly evident in plants infested with *T. urticae*, where roots exhibited a higher number of DEGs at 72 h compared to 24 h (Fig. 3a). A similar response was detected in *Brassica oleracea*, where differential expression peaked in roots at 96 h after infestation by the carterpillar *P. xylostella* [23].

### Foliar herbivory influences how plant hormonal genes are regulated in both leaves and roots, depending on the pest

The feeding mechanism exerted differential effects on the plant hormonal response, leading to distinct responses in leaves and roots, which varied depending on the specific herbivores. JA and ABA responses have been reported to be tightly interconnected, regulating plant defense and stress responses [4, 6, 50]. Leaves infested with *P. brassicae* displayed a greater number of DEGs associated with these hormones. Furthermore, the shared DEGs between *P. brassicae* and *T. urticae* infested leaves showed higher overexpression levels in the former case (Fig. 6a, d). The higher intensity of the stress response elicited by *P. brassicae* infestation under the experiment conditions resulted in modifications to the transcriptomic profile in roots. Plants infested by *P. brassicae* exhibited a greater number of DEGs related to JA and ABA response at 24 h. In contrast, active infestation by *T. urticae* appeared to have a more spatially limited effect, as it slightly altered the expression profile of JA and ABA in roots (Fig. 6a, d). This contrasting pattern in the expression of JA-related genes, depending on the feeding mechanism, has also been observed in other studies, hinting to a greater activation of these pathways in response to the increased foliar damage inflicted by chewing herbivores. In *B. oleraceae* plants, infestation by the chewing caterpillar *P. xylostella* resulted in the upregulation of JA-associated genes in roots, whereas infestation by the sucking aphid *B. brassicae* had no discernible effect on gene expression [23]. Higher activation of JA and ABA responses in plants infested by *P. brassica* hints to a role of these hormones in the short-term response to foliar damage, likely coupled with other stress responses, prioritizing the survival of the plant against an immediate threat.

Conversely, DEGs associated with SA response were more prevalent in leaves infested with *T. urticae*, even at 72 h, after phytophagous removal (Fig. 6b). Previous studies have reported a significant activation of SA responses during *T. urticae* infestation [25, 51], indicating that *T. urticae* likely activates a longer-term induced resistance response, whereas plant response to *P. brassicae* may primarily focus on short-term stress-related processes [52]. This dissimilarity may be caused by the difference in the immediate threat that the plant suffers between both infestations, thereby allowing the plant to activate long-term responses in the infested tissues in the presence of *T. urticae*. Contrastingly, roots exhibited a similar degree of differential expression of SA-related genes across all treatments, even after phytophagous removal (Fig. 6b), suggesting the potential involvement of the SA response in roots during infestation recovery. In roots, the absence of active infestation in this tissue may be causing the lack of disparities between both infestations in contrast to what was observed in leaves.

Despite ET is described to play a role in plant defense mechanisms and is also involved in various biological processes, such as growth, senescence, and stress response [53–55], in this study only a limited number of DEGs related to ET were identified in response to infestation, mostly at 24 h in leaves exposed to phytophagous infestation (Fig. 6c). These DEGs encompassed genes associated with diverse biological processes, including Ethylene Response Factors (Additional file 4), which are known as regulators of multiple cellular functions [56]. Although previous studies have reported changes in the expression of genes involved in ET biosynthesis and ET response in maize (*Zea mays*) upon aboveground herbivory [19], in this study only a reduced number of DEGs associated with ET were observed in roots during and after infestation (Fig. 6c).

Auxin is a well-known primary regulator of plant development [57], but it has also been reported its involvement as a stress regulator [58, 59]. Furthermore, a role for auxin in plant defense against bacterial pathogens and foliar herbivory has been suggested [7, 60, 61]. Leaves infested with *P. brassicae* displayed a higher number of DEGs associated with auxin (Fig. 7a). Although the precise role of auxin in the response against herbivores is still not fully understood, here it appears to participate alongside JA and ABA in the short-term response observed in *P. brassicae* infested leaves. Among auxins, IAA levels have been described to be upregulated in roots of *Nicotiana attenuata* in response to leaf herbivory [22]. At 24 h, DEGs associated with auxin regulation were predominantly observed in roots from *P. brassicae* infested plants, but at 72 h, the number and pattern of DEGs related to auxin regulation was similar between both infestations (Fig. 7a). Hence, it is plausible that auxin response also participates in the prolonged transcriptomic response reported in roots, which aligns with the plant’s recovery after infestation.

Genes associated with other growth-related hormones like CKs and GA also showed a distinct differential expression in leaves actively infested by *P. brassicae* (Fig. 7b, c). These two hormones have crucial roles in plant growth and development, while also participating in the plant defense regulation, interacting with other phytohormones like JA [62, 63]. Furthermore, growth related processes might be particularly affected by the highly intensive stress response caused here by *P. brassicae* feeding and inhibited by the plant defense response as part of a trade-off mechanism with plant growth processes.

### Regulation of flavonoid response and biosynthesis is differentially regulated depending on pest species

Flavonoids, a diverse group of polyphenols synthesized through the phenylpropanoid pathway [64], play a role in stress response, often serving as detoxicants during abiotic stress conditions [65–68], but also in response to pathogens [69, 70]. Previous studies have demonstrated that *P. brassicae* egg deposition leads to the accumulation of flavonols, highlighting the importance of these compounds in establishing the defense response [71]. An enrichment of genes associated with flavonoid biosynthetic process has also been previously described upon *T. urticae* infestation [51]. In leaves, DEGs involved in flavonoid biosynthesis and regulation were predominantly observed in plants infested by *P. brassicae* (Fig. 8), evidencing that various components of the stress response are more active in plants infested by this herbivore. Similarly, a previous meta-analysis with data from *P. rapae* and *T. urticae* infestations reported a higher number of upregulated genes, specially at short times, when leaves were infested by the former [25].

Previous experiments have reported that pest infestation or application of hormones in leaves, like SA, can alter the production of flavonoids in roots [14, 24, 72]. Distinctly, roots from plants infested with *P. brassicae* showed overexpression of genes involved in flavonoid biosynthesis starting at 24 h, while *T. urticae* infested plants displayed DEGs only after infestation, at 72 h (Fig. 8b). Therefore, the upregulation of pathways leading to flavonoid production in roots elicited by these herbivores may resolve possible imbalances created by the consequences of infestation, precising flavonoids as mediators in stress response, antioxidant activity or in root interactions with other organisms [67, 68, 73–75].

### Cell wall components are regulated differentially depending on phytophagous species

The cell wall plays a crucial part in determining the shape and development of plant cells by restricting their ability to elongate or divide [76]. It also has a vital role in plant defense against herbivores, serving a range of roles, from physical barrier to site for receptors involved in plant defense responses [77–80]. Both *P. brassicae* and *T. urticae* infestations resulted in the overexpression of genes involved in cell wall formation and reorganization in leaves, including lignin and pectin, while only roots from plants actively infested with *P. brassicae* exhibited differential expression (Fig. 9a, Additional file 4). Lignin deposition has been previously reported to enhance plant resistance against pathogens [81, 82]. Multiple members of the expansin family, which participate during cell elongation [83, 84], were downregulated during active infestation by *P. brassicae* in leaves (Fig. 9a, Additional file 4). The downregulation of expansin genes suggests that growth-related processes are arrested during infestation by the more physically damaging *P. brassicae*.

In tissues sampled 48 h after phytophagous removal (collection time 72 h), both pests had elicited in roots a downregulation of genes associated with the regulation of pectins, lignins, or hemicelluloses (Fig. 9b). This downregulation profile may be attributed to the plant prioritizing resources for the aerial parts, which had experienced more physical damage from the infestation. Consequently, cell activity in roots might be limited, resulting in arrested growth, and not requiring significant reorganization of the cell wall.

## Conclusions

Our findings corroborated that herbivore infestation triggers distinct molecular changes in the plant dependent on the feeder species, even when the infestation takes place in the same plant species. *P. brassicae* induced a rapid and intense transcriptomic response at 24 h, attributed to the more destructive physical damage inflicted by this chewing-biting herbivore. This response primarily encompassed DEGs associated with short-term stress reactions, including genes linked to JA and ABA responses. In contrast, infested tissues by the piercing-sucking pest *T. urticae* exhibited a longer yet milder transcriptomic response. This response was more persistent after phytophagous removal, involving prevalently DEGs associated with SA response, potentially linked to an induced resistance mechanism. These distinct responses were not confined to the actively infested regions but were also detectable in roots. Roots exhibited an increased number of DEGs at 24 h in *P. brassicae* infested plants, while the prolonged response characteristic of *T. urticae* infested plants was also evident in roots at 72 h. Through the processes differentially affected roots, flavonoid regulation emerged as a significant player in the root reaction to infestation, particularly in response to *P. brassicae*, suggesting their importance in plant defense mechanisms. In summary, this study provides valuable insights into the complex and interconnected responses of plants to herbivore infestation. These findings emphasize the importance of the plant response as a whole, including infested and distal tissues, when analyzing the intricate web of plant defense strategies against different phytophagous species.

### Supplementary Information

**Supplementary material 1.**

**Supplementary material 2.**

**Supplementary material 3.**

**Supplementary material 4.**

## Data Availability

All relevant supporting data sets are included in the article and its supplemental files. RNAseq data have been deposited the European Nucleotide Archive (https://www.ebi.ac.uk/ena/browser/home) and are accessible through the accession number PRJEB70468.

## Abbreviations

ABA: Abscisic acid
CK: Cytokinin
DAMP: Damage-associated molecular pattern
DEG: Differentially expressed gene
ET: Ethylene
GA: Gibberellin
GO: Gene ontology
HAMP: Herbivore-associated molecular pattern
JA: Jasmonic acid
PCA: Principal component analysis
PME: Pectinesterase
RPKM: Reads per kilobase million
SA: Salicylic acid
